# The role of emotional awareness in evaluative judgment: evidence from alexithymia

**DOI:** 10.1038/s41598-023-32242-y

**Published:** 2023-03-30

**Authors:** Rodrigo Díaz, Jesse Prinz

**Affiliations:** 1 Centre for Research in Ethics, University of Montreal, Montreal, Canada; 2 The Graduate Center, CUNY, New York, NY USA

**Keywords:** Psychology, Human behaviour

## Abstract

Evaluative judgments imply positive or negative regard. But there are different ways in which something can be positive or negative. How do we tell them apart? According to Evaluative Sentimentalism, different evaluations (e.g., dangerousness vs. offensiveness) are grounded on different emotions (e.g., fear vs. anger). If this is the case, evaluation differentiation requires emotional awareness. Here, we test this hypothesis by looking at alexithymia, a deficit in emotional awareness consisting of problems identifying, describing, and thinking about emotions. The results of Study 1 suggest that high alexithymia is not only related to problems distinguishing emotions, but also to problems distinguishing evaluations. Study 2 replicated this latter effect after controlling for individual differences in attentional impulsiveness and reflective reasoning, and found that reasoning makes an independent contribution to evaluation differentiation. These results suggest that emotional sensibilities play an irreducible role in evaluative judgment while affording a role for reasoning.

## Introduction

Evaluative judgments do not merely *describe*, but also *evaluate* their targets as good or bad in different ways. For example, “dangerous”, “offensive”, or “foul” (unlike “12ft high”, “1 h late”, or “decayed”) imply negative regard. But each is a different kind of negative regard: Being dangerous is not the same as being offensive, and not the same as being foul. Making evaluative judgments requires distinguishing between different ways in which something can be good or bad. In other words, evaluative judgment requires evaluation differentiation. Someone who confuses beauty and morality wouldn’t make a great art critic, and someone who confuses offense and danger would not make a great moral judge.

To date, little research has investigated evaluation differentiation. How do we distinguish between, for example, offensiveness and dangerousness? To answer this question, we might look at two different theories about the nature of evaluative judgment: Sentimentalism and Rationalism.

According to Sentimentalism, evaluative judgments are grounded on emotion. Some Sentimentalists claim that evaluative judgments *contain* or *express* emotions^1,2^ and thus making an evaluative judgment equals having an emotion or sentiment towards what is being judged. Others claim that evaluative judgments merely *refer to* emotional reactions that would be warranted, but we do not necessarily experience them when making the judgment^3–5^. Disagreements aside, all sentimentalists defend that evaluative judgments *ultimately* depend on humans’ emotional sensibilities, either because evaluative judgments require having an emotion towards what is being judged, or because they require knowing what emotions are warranted by what’s being judged.

Of special importance for our purposes here, Evaluative Sentimentalism posits that each emotion grounds a different type of evaluation. For example, something is *offensive* if it causes or warrants *anger*, and something is *dangerous* if it causes or warrants *fear*. If this is the case, to tell apart offensiveness and dangerousness we need to tell apart anger and fear. Thus, evaluation differentiation requires emotional awareness, i.e., the capacity to recognize emotions in oneself and others.

Against the Sentimentalist view, some argue that evaluative judgment is a matter of dispassionate reasoning. According to Evaluative Rationalism, evaluative judgments are grounded on objective features of the things we judge^6,7^. This way, whether something is offensive or dangerous depends on its particular characteristics, and not on our emotions towards it. According to Rationalism, something is offensive if it violates a social rule, and something is dangerous if it has the potential to cause injury. Emotions appear nowhere in these definitions. Thus, evaluation differentiation does not require emotional awareness. Instead, it requires reasoning from non-emotional facts to conclusions about their evaluative import.

Both Sentimentalism and Rationalism offer plausible views about the psychological mechanisms underlying evaluation differentiation. According to Sentimentalism, evaluation differentiation is a matter of emotional awareness. According to Rationalism, evaluation differentiation is a matter of inferential reasoning and attention to non-emotional features of what's being judged. Here, we provide an empirical test of Evaluative Sentimentalism by investigating the relation between alexithymia (a deficit in emotional awareness) and evaluation differentiation. If Evaluative Sentimentalism is true, people who are less skilled at distinguishing emotions should also have problems distinguishing evaluations. We found support for this prediction in two separate studies.

### Previous studies on emotion and evaluative judgment

Most work on emotion and evaluative judgment has focused on one particular type of judgment: moral judgment^2,8^. Numerous studies indicate that experimentally induced emotions^9–11^, trait emotions^12,13^, and emotional impairments^14–16^ impact participants’ judgments of moral wrongness or acceptability.

Outside the moral domain, a wealth of research has reported mood-congruency effects on evaluative judgment^17,18^. That is, the tendency to make evaluative judgments that are consistent with one’s positive or negative emotional state. This research shows that people who are in a good mood tend to make more positive judgments of objects or persons, while people who are in a bad mood tend to be more negative in their evaluations.

Previous work on emotion and moral judgment and mood-congruency effects suggest that evaluative judgments are grounded on emotion, as Evaluative Sentimentalism posits^19–21^. Most of these studies did not test whether different emotions impact different types of evaluative judgment. There are, however, a few exceptions to this rule.

According to the CAD hypothesis^22^, different types of moral violations are associated with different emotions. In particular, contempt is linked to community violations, anger is linked to autonomy violations, and disgust is linked to purity violations^23^. In line with this idea, some studies have found that anger preferentially impacts moral judgments concerning violations of individual rights, and disgust preferentially impacts moral judgments of “impure” behavior^22,24–27^. Arguably, violations of individual rights tend to be considered offensive, and violations of purity tend to be considered foul. Thus, although these studies didn’t ask participants to make different types of evaluations, they suggest that anger is related to judgments of offensiveness and disgust is linked to judgments of foulness, as Evaluative Sentimentalism claims.

Another well-studied emotion-evaluation link is the one between fear and dangerousness. Studies have shown that experimentally-induced fear^28^, trait fear^29^, and fear impairments^30,31^ impact participants’ judgments of danger and, importantly, that fear impacts judgments of danger in a different way than other negative emotions^32^. These results support the Sentimentalist idea that different emotions ground different types of evaluations. In particular, Evaluative Sentimentalism posits that humans’ capacity to judge things as dangerous is grounded on their capacity to experience fear. If this is the case, it comes as no surprise that individuals’ fear propensities and (lack of) fear experiences have an impact on their judgments of danger.

The studies reviewed in this section fit the core tenets of Evaluative Sentimentalism, according to which evaluative judgments are grounded on emotion, and different emotions ground different types of evaluation. However, no study to date has directly tested Evaluative Sentimentalism’s predictions regarding evaluation differentiation. Namely, that evaluation differentiation requires emotional awareness or, more specifically, the ability to distinguish between different emotions.

### The present research

To explore the impact of emotional awareness on evaluation differentiation, we will look at individual differences in alexithymia. The word “alexithymia” etymologically means “no words for emotion”^33^. In contemporary research, alexithymia is understood as a cluster of deficits in emotional awareness including difficulties identifying, describing, and thinking about emotions^34,35^. Each of these three dimensions is measured by a subscale of the 20-item Toronto Alexithymia Scale^36^, which is the most widely used measure of alexithymia and emotional awareness more generally^37^.

There are inconsistent findings on whether high alexithymia individuals have impaired emotional experience^38^^.^ However, what matters for our purposes here is emotional awareness, regardless of whether emotional experience is intact or not. Remember that not every Sentimentalist view is committed to the claim that making an evaluative judgment requires having an emotion at the moment of judging, but they all share the idea that evaluation differentiation requires emotional awareness (see Introduction).

High alexithymia individuals have problems identifying emotions in themselves and others. While they can distinguish between positive and negative feelings, they have problems making more fine-grained distinctions between emotions^39^. If, as Evaluative Sentimentalism claims, different emotions ground different types of evaluation, high alexithymia individuals should not only have problems distinguishing emotions, but also have problems distinguishing evaluations. This motivates our main hypothesis:

#### H1

Higher alexithymia is related to lower evaluation differentiation.

It is important to note that H1 does not sit well with Evaluative Rationalism. Rationalism is compatible with individuals sometimes using their emotions as input to make evaluative judgments^40^, but it denies that emotions (or considerations about emotions) are required for evaluative judgment. Individuals high in alexithymia cannot identify their emotions, but they can make evaluative judgments based on non-emotional sources of information. Thus, if Rationalism is true, higher alexithymia should not impact evaluation differentiation.

Previous research suggests that high alexithymia individuals have the most problems distinguishing between anger, fear, disgust, and sadness^41^. According to Evaluative Sentimentalism, these emotions ground judgments of offense, danger, foulness, and loss, respectively. Thus, our studies will test the impact of alexithymia on participants’ ability to distinguish between these particular evaluations.

Despite the recent surge in research on people’s ability to distinguish emotions^42–46^, no study to date has examined its relation with evaluation differentiation. However, a few studies have indicated an impact of alexithymia on moral judgment^47,48^ and judgments of danger^49^, suggesting that emotional awareness impacts evaluative judgment.

## Study 1

In order to test H1, Study 1 recorded participants' evaluations of a series of affective pictures, as well as their alexithymia levels. Because we also wanted to confirm the received view that high alexithymia is related to low emotion differentiation, we recorded another group of participants’ alexithymia levels and emotional reactions to the same pictures. We consider four emotions (fear, anger, disgust, sadness) and their associated evaluations (danger, offense, foulness, loss).

Note that we are interested in the association between participants’ trait emotional awareness and their ability to distinguish evaluations, and not in the association between participants’ emotions and evaluations about the same stimuli.

### Methods

151 participants were recruited through Amazon Mechanical Turk and completed the survey for a monetary payment. 3 participants didn’t pass the attention check (see below) leaving a final sample of 148 participants (93 male, 55 female, Mage = 37.45, SD = 11.40, age-range 18–70). Sensitivity analyses using G*Power showed that, with at least 72 participants per group, there was enough statistical power to detect a medium-sized effect of r = 0.4 using bivariate correlation. With two groups of 15 and 47 participants respectively, we had enough power to detect an effect of d = 1.01 using Wilcoxon-Mann–Whitney test.

Participants were presented with a series of 12 pictures from the Nencki Affective Picture System (NAPS)^50^. Following previous normative ratings^51^, we selected pictures that tend to elicit a single distinguishable emotion. In particular, 3 fear pictures (e.g., a snake), 3 anger pictures (e.g., a violent scene), 3 disgust pictures (e.g., a dirty fridge), and 3 sadness pictures (e.g., a car accident). Each picture was presented for 6 s. The order of presentation was counterbalanced.

Participants were randomly assigned to one of two groups (Emotion, Evaluation). Participants in the Emotion group rated to what extent each of the 12 pictures made them feel (1) “Afraid”, (2) “Angry”, (3) “Grossed out” and/or (4) “Sad” using scales from 0 (“Not at all”) to 100 (“Extremely”). Participants in the Evaluation Group rated to what extent they evaluate what they saw in the pictures as (1) “Dangerous”, (2) “Offensive”, (3) “Foul” and/or (4) “Irrevocable loss” using scales from 0 (“Not at all”) to 100 (“Extremely”).

Finally, participants filled out the Toronto Alexithymia Scale (TAS-20)^36^. This scale consists of 20 items classified into three dimensions: Difficulty Identifying Feelings (DIF, α = 0.937), Difficulty Describing feelings (DDF, α = 0.836), and Externally Oriented Thinking (EOT, α = 0.612). The scale included an attention check (“When answering this question, I choose ‘very much’ to let researchers know that I’m paying attention”) which was used as a participant selection criteria.

Following previous work^52,53^, we computed Emotion differentiation and Evaluation differentiation scores as the across-pictures average of the distance between participants’ highest-rated emotion/evaluation and the other emotions/evaluations divided by the mean intensity of their ratings:

$${\text{Differentiation}}\, = \,{\text{Mean }}[\left( {{\text{Highest }}\;{\text{Rating}}{-}{\text{Mean}}\; \, \left( {{\text{Lower}}\;{\text{ Ratings}}} \right)} \right) \, /{\text{ Mean }}\left( {{\text{All }}\;{\text{Ratings}}} \right)]$$

The reason to divide the distance between ratings by their intensity is that the same distance can indicate more or less differentiation depending on the intensity of the ratings. For example, a distance between ratings of 100 and 80 indicate less differentiation than a distance between ratings of 40 and 20. Dividing distance by average intensity addresses this issue.

### Results

Kolmogorov–Smirnov test indicated that none of our outcome variables follow a normal distribution (all *p*s < 0.011). Thus, statistical analyses use Spearman’s Rho for correlations and Mann–Whitney U for comparisons between groups. Descriptive statistics and correlations between all variables can be found in Table 1.

**Table 1:** Correlation coefficients (Spearman’s ρ) and descriptive statistics (mean and standard deviation) for all variables in Study 1.

	Mean (SD)	EmDiff	EvDiff	DIF	DDF	EOT	TAS
EmDiff	1.58 (.85)	1		− .521**	− .383*	− .148	− .422**
EvDiff	1.51 (.91)		1	− .419**	− .282*	− .390**	− .416**
DIF	13.90 (7.50)			1	.793**	.423**	− .872**
DDF	11.41 (4.92)				1	.484**	− .888**
EOT	19.68 (4.89)					1	.746**
TAS	44.99 (14.68)						1

* indicates *p* < .05, ** indicates *p* < .001.

**Figure 1:**
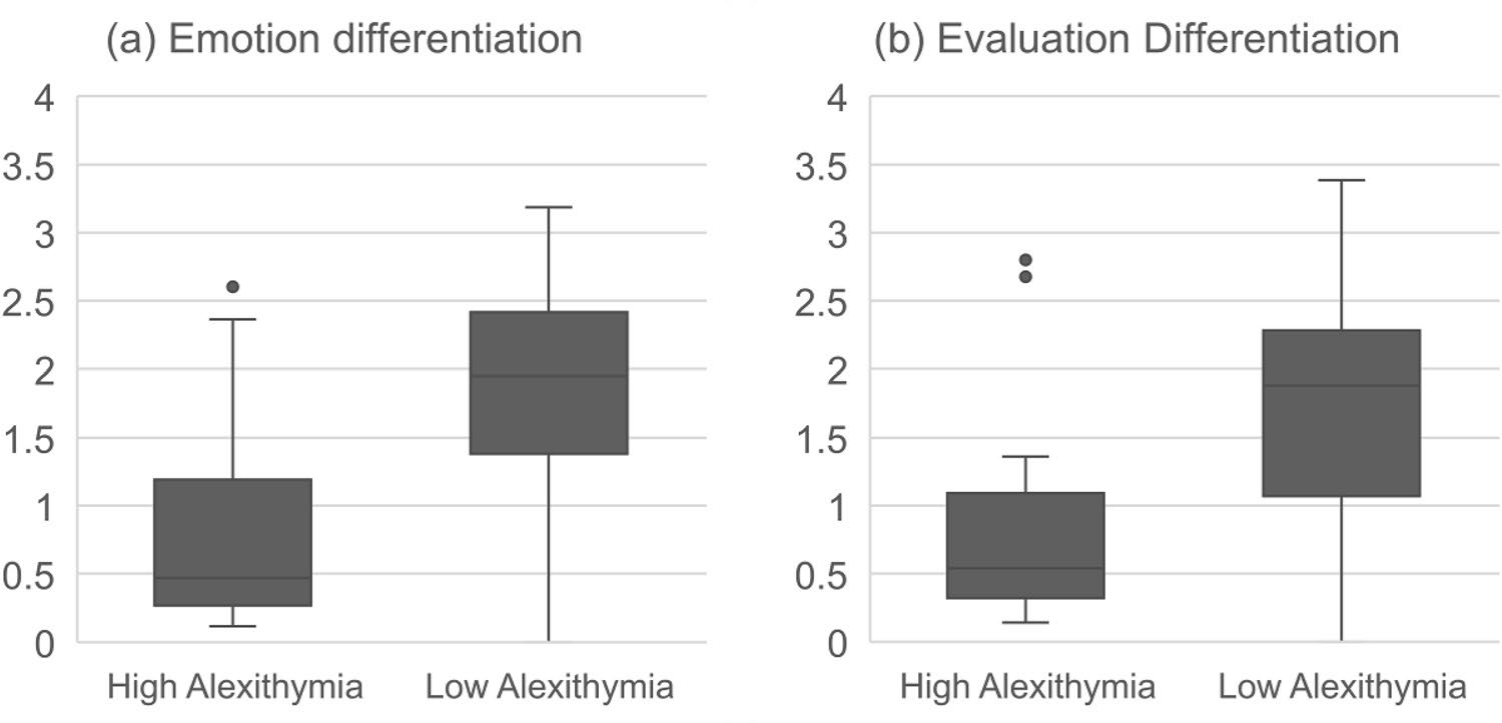
Boxplot for (**a**) Emotion Differentiation, and (**b**) Evaluation differentiation scores by alexithymia groups (High vs. Low) in Study 1. Low scores mean that participants’ ratings tended to be similar across different emotions (fear, anger, disgust, sadness) or evaluations (dangerous, offensive, foul, irrevocable loss).

Comparisons between High Alexithymia (TAS > 60, n = 15) and Low Alexithymia (TAS < 52, n = 47–51) groups of participants ^54,55^ were computed. There were significant differences between the High Alexithymia and Low Alexithymia groups for both Emotion Differentiation, *U* = 135, *z* = − 3.79, *p* < 0.001, *d* = 1.05, and Evaluation Differentiation, *U* = 144, *z* = − 3.43, *p* = 0.001, *d* = 0.967 (see Fig. 1).

### Discussion

The results of Study 1 suggest that alexithymia is not only related to difficulties differentiating emotions (as previous research has found) but also to difficulties differentiating evaluations (supporting H1).

In response, one could argue that both difficulties are driven by a third factor: reasoning. Critics of Sentimentalism have claimed that extant results regarding the impact of emotion on evaluative judgment can be explained in terms of emotions’ influence on reasoning and attention^56–60^. If people high in alexithymia fail to pay attention to the relevant features of what they are judging, or fail to infer what those features entail, their problems differentiating evaluations could be explained in terms of impaired reasoning. In order to explore this alternative explanation, we reran Study 1 adding measures of reflective reasoning and attentional impulsivity.

## Study 2

Study 2 aims to replicate and extend the findings from Study 1. Design and analysis plans were preregistered (https://osf.io/khe3r/?view_only=d3b2efb89c9a4b939d594a37b704bde5). The main addition is the inclusion of individual difference measures of attentional impulsiveness and reflective reasoning. This allows us to test Rationalist alternatives to Sentimentalism. Thus, in addition to H1 (see Introduction: The present research), we preregistered the following rationalist prediction:

### H2

Higher Attentional Impulsiveness is related to lower Evaluation differentiation / Higher Reflective Reasoning is related to higher Evaluation differentiation.

H1 and H2 are compatible and independently plausible. However, if the effect of alexithymia disappears after controlling for individual differences in attention and reasoning, this would mean that the results of Study 1 could be explained away in terms of dispassionate reasoning (against H1).

### Method

301 participants were recruited through Prolific Academic and completed the survey for a monetary payment. 4 participants didn’t pass the attention check (see below) and 1 participant mistook the instructions of one of our scales as an attention check leaving a final sample of 296 participants (132 male, 162 female, 2 non-binary, M_age_ = 34.41, SD = 13.17, age-range 18–80). Sensitivity analyses using G*Power showed that there was enough statistical power to detect an effect of r = 0.2 using bivariate correlation, and f2 = 0.04 using multiple regression with 5 predictors. With two groups of 31 and 218 participants respectively, we had enough power to detect an effect of d = 0.65 using a Wilcoxon-Mann–Whitney test.

Participants were presented with the same pictures used in Study 1, the same evaluation questions, and the same alexithymia scale (TAS-20) with subscales for Difficulty Identifying Feelings (DIF, α = 0.862), Difficulty Describing feelings (DDF, α = 0.797) and Externally Oriented Thinking (EOT, α = 0.524).

Afterwards, participants filled out the Attentional Impulsiveness scale^61^, which consists of 5 items (e.g. “I don’t pay attention”, α = 0.818) measuring participants’ lack of attention.

Finally, participants completed the Cognitive Reflection Test^62,63^, which consists of three logical, verbal, and arithmetic reasoning problems varying in difficulty (α = 0.706). Each participant received a score from 0 to 3 according to the number of correct responses.

### Results

Kolmogorov–Smirnov test indicated that our outcome variable didn’t follow a normal distribution (*p* = 0.005). Thus, statistical analyses use Spearman’s Rho for correlations, Mann–Whitney U for comparisons between groups, and bootstrapped multiple regression. Descriptive statistics and correlations between all variables can be found in Table 2.

**Table 2:** Correlation coefficients (Spearman’s ρ) and descriptive statistics (mean and standard deviation) for all variables in Study 2.

	Mean (SD)	EvDiff	AI	CRT	DIF	DDF	EOT	TAS
EvDiff	1.67 (.65)	1	− .051	.172*	− .126*	− .087	− .166*	− .163*
AI	9.49 (3.20)		1	.075	.443**	− .472**	.337**	.514**
CRT	1.42 (1.18)			1	.004	.022	.029	.023
DIF	13.74 (5.77)				1	.723**	.207**	.851**
DDF	12.24 (4.61)					1	.307**	.865**
EOT	18.9 (4.35)						1	.597**
TAS	44.87 (11.58)							1

* indicates *p* < .05, ** indicates *p* < .001.

**Figure 2:**
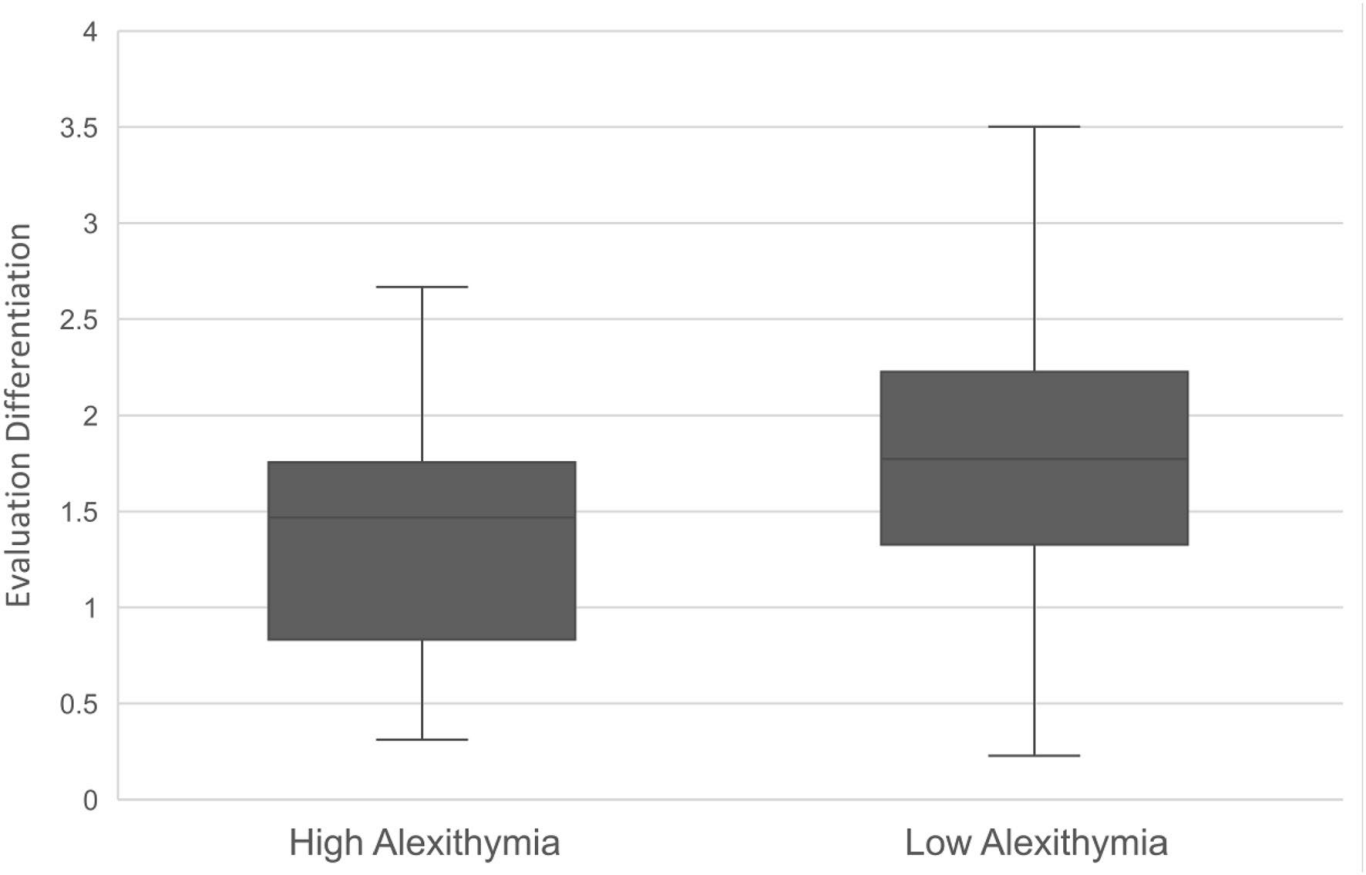
Boxplot for Evaluation differentiation scores by Alexithymia groups (High vs. Low) in Study 2. Low scores mean that participants’ ratings tended to be similar across different emotions (fear, anger, disgust, sadness) or evaluations (dangerous, offensive, foul, irrevocable loss).

There was a significant difference between the High Alexithymia (TAS > 60, n = 31) and Low Alexithymia (TAS < 52, n = 218) groups for Evaluation Differentiation scores, *U* = 2390, *z* = − 2.64, *p* = 0.008, *d* = 0.339 (see Fig. 2).

Bootstrapped multiple linear regression analyses using Evaluation differentiation as outcome variable and DIF, DDF, EOT, AI, and CRT scores as predictors showed significant effects of Difficulty Identifying Feelings (DIF), Externally Oriented Thinking (EOT), and Reflective Reasoning (CRT). Results are detailed in Table 3.

**Table 3:** Bootstrapped multiple linear regression model predicting Evaluation Differentiation scores (Study 2).

	B	b.c. 95% CI	t	*p*	sr
Constant	1.815	[1.557, 2.062]	13.610	.000	
AI	.011	[− .015, 038]	.828	.405	.047
CRT	.090	[.024, .160]	2.916	.002	.164*
DIF	− .021	[− .041, − .003]	− 2.360	.021	− .133*
DDF	.010	[− .012, .030]	.854	.378	.048
EOT	− .027	[− .046, − .010]	− 3.015	.003	− .170*
R^2^ / Adj. R^2^	.082 / .066				

* indicates *p* < .05. Significant values are in bold.

### Discussion

Replicating the results of Study 1, we found a significant association between alexithymia levels and evaluation differentiation. In particular, participants’ difficulty identifying feelings (DIF) and tendency to avoid thinking about emotions (EOT) were related to lower evaluation differentiation. Crucially, these associations remained significant after controlling for individual differences in attentional impulsiveness and reflective reasoning. This provides further support for Evaluative Sentimentalism (H1).

Supporting H2, we found a significant association between reflective reasoning and evaluation differentiation. However, reflective reasoning was not significantly correlated with alexithymia. Thus, reasoning abilities cannot explain away the association between alexithymia and evaluation differentiation. Instead, the results suggest that emotional awareness and reflective reasoning have independent effects on participants’ ability to distinguish evaluations. Attentional impulsiveness, on the other hand, was significantly correlated with alexithymia, but not with evaluation differentiation. Thus, it seems like high alexithymia individuals’ also lack attention to non-emotional features of their environment, but this does not explain their problems in evaluative judgment differentiation.

## General discussion

Evaluative Sentimentalism claims that evaluative judgments are grounded on emotion, and different emotions ground different types of evaluation. It follows that people who are less skilled at distinguishing emotions should also have problems distinguishing evaluations. We confirmed this prediction in two studies, using alexithymia as a measure of emotional awareness. Study 1 found that high alexithymia is not only related to low emotion differentiation, but also low evaluation differentiation. Study 2 replicated this effect after controlling for individual differences in attentional impulsivity and reflective reasoning, and found that reasoning makes an independent contribution to evaluation differentiation. Overall, our results suggest that emotional awareness plays an irreducible role in evaluation differentiation. To tell whether something is offensive, dangerous, foul, or a loss, we need to distinguish between anger, fear, disgust, and sadness. This supports the Sentimentalist’s picture of evaluative judgment.

Some limitations to our results are worth noting. First, our studies tested only a limited number of evaluative judgments (dangerousness, offensiveness, foulness, and loss). We selected these evaluations based on the specific deficits involved in alexithymia (see Introduction: The present research). However, future studies should investigate whether our results can be extended to a wider range of evaluations. Evaluative Sentimentalism claims that each emotion kind grounds a particular type of evaluation. Future studies could test, for example, whether aesthetic appreciation grounds judgments of beauty, or whether indignation grounds judgments of unfairness. These investigations would help us determine the scope of Evaluative Sentimentalism.

Second, the results of Study 2 suggest that both reasoning and emotion contribute to evaluation differentiation. Even though reasoning doesn’t explain away the role of emotion, our results prompt us to make room for reasoning processes in our theories of evaluative judgment. There are different ways of doing so. One possibility is that reasoning and emotion interact. For example, reasoning might help refine our emotional sensibilities, and emotional experiences might feed into reasoning processes^64,65^. Another possibility is that reasoning and emotion provide separate routes. Under this “dual-process” view, our evaluative judgments might be sourced on emotion or reasoning depending on the context^66–68^.

Third, one might worry that the predictive value of the regression model in Study 2, and the effect of difficulty identifying feelings in particular, is too low to provide support for Evaluative Sentimentalism. It is important to note that, if Evaluative Sentimentalism is false, there is no reason to expect an effect of difficulty identifying feelings on evaluative differentiation (see Introduction: The present research). Thus, a small effect already supports the Sentimentalist picture. But why was the effect relatively low? One potential explanation is that individuals high in alexithymia do not completely lack the ability to distinguish emotions. The results of Study 1 support this idea.

We hope that our results pave the way for future investigations of the multifarious nature of evaluative judgment, as well as the interplay between emotion and reasoning in evaluation differentiation.

### Ethics approval

This research was approved by the Research Ethics Committee at the University of Montreal (CERSC-2021-008-D). All methods were carried out in accordance with relevant guidelines and regulations. Informed consent was obtained from all participants.

## Data Availability

Data and materials are available at https://osf.io/y9hgj/?view_only=ace13d2f454f48cbaa7f01d56f0ba159.
